# The value of phenylalanine in predicting atrial fibrillation risk in chronic heart failure

**DOI:** 10.3389/fcvm.2024.1392548

**Published:** 2024-08-20

**Authors:** Qing-Fen Zhou, Qiu-Ya Lu, Yang Dai, Qiu-Jing Chen, Xiao-Shuang He, Shuai Chen, Jun-Tao Zhao, Feng-Ru Zhang, Lin Lu, Fan Yang

**Affiliations:** ^1^ Department of Cardiovascular Medicine, Rui Jin Hospital, Shanghai Jiao Tong University School of Medicine, Shanghai, China; ^2^ Department of Clinical Laboratory, Rui Jin Hospital, Shanghai Jiao Tong University School of Medicine, Shanghai, China; ^3^ Institute of Cardiovascular Disease, Shanghai Jiao Tong University School of Medicine, Shanghai, China; ^4^ Department of Pharmacy, Rui Jin Hospital, Shanghai Jiao Tong University School of Medicine, Shanghai, China

**Keywords:** chronic heart failure, atrial fibrillation, phenylalanine, risk prediction, inflammatory disorder, prognosis

## Abstract

**Backgrounds:**

Atrial fibrillation (AF) is a common complication of chronic heart failure (HF). Serum phenylalanine (Phe) levels are related to inflammation disorder. It is meaningful to study the circulating Phe with AF occurrence in HF.

**Methods:**

The cross-sectional study recruited 300 patients (78.0% male; mean age, 65 ± 13 years) with HF (left ventricular ejection fraction of ≤50%, containing 70 AF patients) and 100 normal controls. Serum Phe value was measured by liquid chromatography–tandem mass spectrometry. Logistic regression analysis was conducted to measure the association between Phe and AF risk in HF. The association between Phe and high-sensitivity C-reactive protein (hsCRP) was assessed by simple correlation analysis. In the prospective study, the 274 HF subjects (76.6% male; mean age, 65 ± 13 years) were followed up for a mean year (10.99 ± 3.00 months).

**Results:**

Serum Phe levels increased across the control, the HF without AF, and the HF with AF groups (77.60 ± 8.67 umol/L vs. 95.24 ± 28.58 umol/L vs. 102.90 ± 30.43 umol/L, ANOVA *P* < 0.001). Serum Phe value was the independent risk factor for predicting AF in HF [odds ratio (OR), 1.640; 95% CI: 1.150–2.339; *P* = 0.006]. Phe levels were correlated positively with hsCRP value in HF patients with AF (*r* = 0.577, *P* < 0.001). The elevated Phe levels were associated with a higher risk of HF endpoint events in HF patients with AF (log-rank *P* = 0.005).

**Conclusions:**

In HF with AF subjects, elevated Phe value confers an increased risk for prediction AF and was more related to poor HF endpoint events. Phe can be a valuable index of AF in HF.

## Introduction

Chronic heart failure (HF) is a major public health problem worldwide with a high hospitalization rate and huge economic burden (1, 2). In HF patients, many mechanisms, including maladaptive gene expression, structural remodeling, inflammation, and oxidative stress, predispose to the progression of atrial fibrillation (AF) (3, 4). AF is a common complication of HF with an average prevalence of 25% (5), which can also exacerbate HF. Moreover, several kinds of research have shown that HF and AF share common pathophysiological mechanisms and often coexist. When both are present, the interaction of physiological processes makes each condition more likely to progress (6). In addition, the co-existence of HF and AF has been found to increase the risk of stroke, hospitalization for worsening HF, and all-cause mortality (7). Thus, it is detrimental to HF patients when complicated with AF. However, there remains a lack of circulatory markers to diagnose and predict the incidence of AF in HF patients.

Amino acids (AAs) play a key role in nutrient metabolism necessary for cellular survival and development (8). In a large prospective cohort study, Würtz P et al. identified that serum phenylalanine (Phe) level may be a biomarker for cardiovascular (CV) risk (9). Further research showed that Phe levels were sensitive to HF lesions by inflammation responses (10). Meanwhile, the increased plasma concentration of Phe and disrupted Phe metabolism predict poor outcomes in critical patients phenotypically, predominantly those presenting with HF (11, 12). However, the association between Phe and the comorbidity of HF has not been further studied. Considering the worse prognosis in HF when combined with AF (13), we designed this study to investigate the correlation between Phe and AF in chronic HF patients, in search of a potential biomarker of AF occurrence for better management of HF disease.

## Methods

This study was approved by the institutional review committee of Ruijin Hospital, Shanghai Jiao Tong University School of Medicine, Shanghai, China, and was conducted in accordance with the tenets of the 1975 Declaration of Helsinki. Written informed consent was obtained from all participants prior to enrollment.

This study was designed as two separate sets for analysis. The cross-sectional analysis was performed on a total of 300 HF patients (78.0% male; mean age, 65 ± 13 years) to explore the correlation between serum Phe levels and the occurrence of AF. The prospective analysis was performed on 274 HF patients (76.6% male; mean age, 65 ± 13 years) to examine the prognostic value of Phe.

### Study population

The study included 337 consecutive patients with stable HF who had been using diuretics for at least 3 months and had been hospitalized for HF at least once in the past year. To be eligible for the study, patients had to have N-terminal pro-brain natriuretic peptide (NT-proBNP) levels of over 300 pg/ml for those with a normal heart rhythm and 900 pg/ml for those with AF at the beginning of the study. All participants were men and women aged 18 years or older. However, patients with recent acute coronary syndrome, significant concomitant diseases such as infection or autoimmune disease, recent myocardial infarction, stroke, open-heart surgery within the past 4 weeks, mechanical ventilation, renal replacement therapy, or postcardiac transplantation were excluded from the study. A total of 318 patients with symptomatic and echocardiographically confirmed chronic HF (ejection fraction, ≤50%) were provisionally recruited. Eighteen patients without an electrocardiogram (ECG) or 24-h ambulatory electrocardiogram (Holter) report were also excluded due to the inability to confirm their heart rhythm (14). The final cohort of HF patients (*n* = 300) was divided into three groups based on the tertiles of Phe value: the low-Phe group (Phe ≤ 80.94 umol/L, *n* = 100), the intermediate-Phe group (81.07 umol/L ≤ Phe ≤103.97 umol/L, *n* = 100), and the high-Phe group (Phe ≥ 104.05 umol/L, *n* = 100). Events of AF, including paroxysmal, persistent, and permanent AF, were acquired by reviewing the patient's medical history and then confirmed by ECG or Holter report. Ultimately, 70 of the 300 patients with HF were confirmed to have comorbid AF (non-valvular AF). In addition, we collected 100 healthy controls who were age- and gender-matched to the HF patients.

### Follow-up and outcomes

The HF endpoints in this study focused on cardiovascular death and hospitalization due to HF. Follow-up surveys were conducted either in person at hospitals or over the phone. To ensure accuracy, all endpoints were verified by independent cardiologists. The recurrence of acute HF in patients with chronic HF was diagnosed based on various factors including signs, symptoms, abnormal lab results, and imaging tests.

### Clinical assessments, biochemical tests, and measurements of amino acids

Comprehensive demographic information and clinical characteristics were gathered from all patients. Body mass index (BMI) was determined using the formula weight/height^2^ in kilograms per square meter. Blood pressure (BP) was assessed on the arm while the patient was seated after resting for 10 min. Hypertension was diagnosed in accordance with the guidelines stated in The Seventh Report of the Joint National Committee on Prevention, Detection, Evaluation, and Treatment of High Blood Pressure (JNC7) (15).

Blood samples were collected following an overnight fast for laboratory analysis. Plasma levels of creatinine, triglycerides, and total cholesterol were measured using the Hitachi 912 Analyzer from Roche Diagnostics in Germany. The estimated glomerular filtration rate (eGFR) was calculated using the Chronic Kidney Disease Epidemiology Collaboration (CKD-EPI) equation (16). Renal dysfunction was defined as an eGFR at or below 60 ml/min/1.73 m^2^. Glycosylated hemoglobin (HbA1c) levels were assessed using ion-exchange high-performance liquid chromatography equipment from Bio-Rad Laboratories in the USA. Alanine transaminase (ALT) levels were analyzed using the Beckmann AU5821 automatic biochemical analyzer and compatible reagents. NT-proBNP levels were measured with a commercially available ELISA kit from Roche Diagnostics. High-sensitivity C-reactive protein (hsCRP) levels were determined using an ELISA kit from Biocheck Laboratories in Toledo, OH, USA.

Serum Phe levels were analyzed using mass spectrometry with the Recon amino acid kit containing 21 items, conducted on the Recon RZ500 instrument (17). The RZ500 mass spectrometer was developed in collaboration between Shanghai Reigncom Biotechnology Co., Ltd. (China) and Thermo Fisher Scientific, offering performance comparable to Thermo Fisher's high-end Thermo Scientific TSQ Altis triple quadrupole mass spectrometer. The 21-amino acid assay kit is a medical device registered in China by Reigncom in 2021 and obtained the CE-IVD mark in 2023.

### Electrocardiogram and echocardiographic examination

Upon admission, an initial ECG was performed using the Beijing Madix MECG-300 12-lead handheld acquisition recorder, calibrated with standard settings at a speed of 25 mm/s and a voltage of 10 mm/mV. Holter monitoring was conducted using the Schiller medilogAR EC-3H device with three channels for a duration of 24 h. Data from ECG and Holter recordings were extracted from the system and independently analyzed by two experienced cardiologists. Two-dimensional (2D) echocardiography and Doppler flow imaging were carried out on the second day post-admission. The imaging was captured using the GE Vivid-I system with a 1.9- to 3.8-MHz phased-array transducer, recording images from standard parasternal, and apical four-chamber views at a frame rate of 60–100 frames/s. Digital data were analyzed offline using EchoPAC version 7 software by two cardiologists who were blinded to the laboratory results. Measurements such as left ventricular end-diastolic diameter, left atrial diameter (LAD), aortic dimensions, interventricular septal thickness, and left ventricular posterior wall thickness were obtained via M-mode echocardiography. Left ventricular ejection fraction (LVEF) was calculated using Simpson's biplane method on two-dimensional apical four-chamber views.

### Statistical analysis

Continuous data were presented as mean ± standard deviation (SD), while categorical data were shown as frequencies or percentages. The normality of continuous variables was assessed using the Kolmogorov–Smirnov test, and transformations such as logarithmic or square root were applied to variables with non-normal distributions. Chi-squared tests were utilized for comparing categorical variables. To analyze trends among groups, one-way ANOVA was employed, followed by *post hoc* comparisons using the least significant difference (LSD) procedure. The patients were categorized into Phe tertiles, and logistic regression analysis was conducted to determine odds ratios (ORs) with 95% CIs regarding the association between Phe levels and AF risk in HF patients. Log-transformed Phe levels were examined as continuous variables. Two models were considered for adjustment: Model 1 included age and gender, while Model 2 (full adjustment) additionally adjusted for factors such as BMI, systolic BP, smoking, drinking, diabetes mellitus (DM), hyperlipidemia, coronary disease, LAD, log NT-proBNP, low eGFR, and hsCRP. Receiver-operating characteristic (ROC) curve analysis was performed based on logistic regression predictions to determine the optimal cutoff values and the area under the curve (AUC). The association between serum Phe levels and hsCRP markers was assessed through simple correlation. Then, they were further verified by logistic regression analyses adjusted for age, gender, BMI, log troponin-I, log NT-proBNP, hbA1c, low-density lipoprotein cholesterol (LDL-c), ALT, and low eGFR. Kaplan–Meier curves were used to ascertain event-free survival, with log-rank tests determining differences in survival across Phe tertiles. Statistical analysis was conducted using SPSS 22.0 software, considering a significance level of *P* < 0.05.

## Results

### Clinical characteristics

The comparison of clinical features and hematological indicators between the HF and control groups is presented in Table 1. The controls and HF patients did not differ significantly with respect to gender, age, and total cholesterol level (*P* > 0.05). The control group had no history of AF, coronary disease (CAD), hypertension, hyperlipidemia, stroke, DM, or chronic kidney disease (CKD). Compared to the controls, those with HF showed decreased levels of high-density lipoprotein cholesterol (HDL-c) and eGFR, as well as elevated levels of triglycerides, LDL-c, fasting blood glucose, ALT, and Phe. (*P* < 0.05).

**Table 1:** Baseline characteristics of all subjects.

	Control	HF	*P* value
	*n* = 100	*n* = 300	
Clinical features			
Men, *n* (%)	74 (74.0)	234 (78.0)	0.410
Age (years)	63 ± 13	65 ± 13	0.397
NYHA class	1.00 ± 0.00	2.74 ± 0.68	<0.001
Medical history			
AF, *n* (%)	0 (0.0)	70 (23.3)	<0.001
Coronary disease, *n* (%)	0 (0.0)	168 (56.0)	<0.001
Hypertension, *n* (%)	0 (0.0)	186 (62.0)	<0.001
Hyperlipidemia, *n* (%)	0 (0.0)	19 (6.3)	0.010
Stroke, *n* (%)	0 (0.0)	15 (5.0)	0.023
Diabetes mellitus, *n* (%)	0 (0.0)	106 (35.3)	<0.001
Chronic kidney disease, *n* (%)	0 (0.0)	35 (11.7)	<0.001
Laboratory values			
Triglyceride (mmol/L)	0.92 ± 0.21	1.34 ± 0.70	<0.001
Total cholesterol (mmol/L)	4.18 ± 0.53	4.05 ± 1.18	0.138
HDL-c (mmol/L)	1.77 ± 0.32	1.09 ± 0.27	<0.001
LDL-c (mmol/L)	2.19 ± 0.39	2.47 ± 1.01	<0.001
FBG (mmol/L)	5.30 ± 0.21	6.42 ± 2.70	<0.001
ALT (IU/L)	15.10 ± 3.72	32.02 ± 59.27	<0.001
eGFR (ml/min/1.73 m^2^)	86.33 ± 12.15	66.99 ± 25.77	<0.001
Phenylalanine (umol/L)	77.60 ± 8.67	97.03 ± 29.15	<0.001

HF, heart failure; NYHA New York Heart Association; AF, atrial fibrillation; HDL-c, high-density lipoprotein cholesterol; LDL-c, low-density lipoprotein cholesterol; FBG, fasting blood glucose; ALT, alanine transaminase; eGFR, estimated glomerular filtration rate.

**Figure 1:**
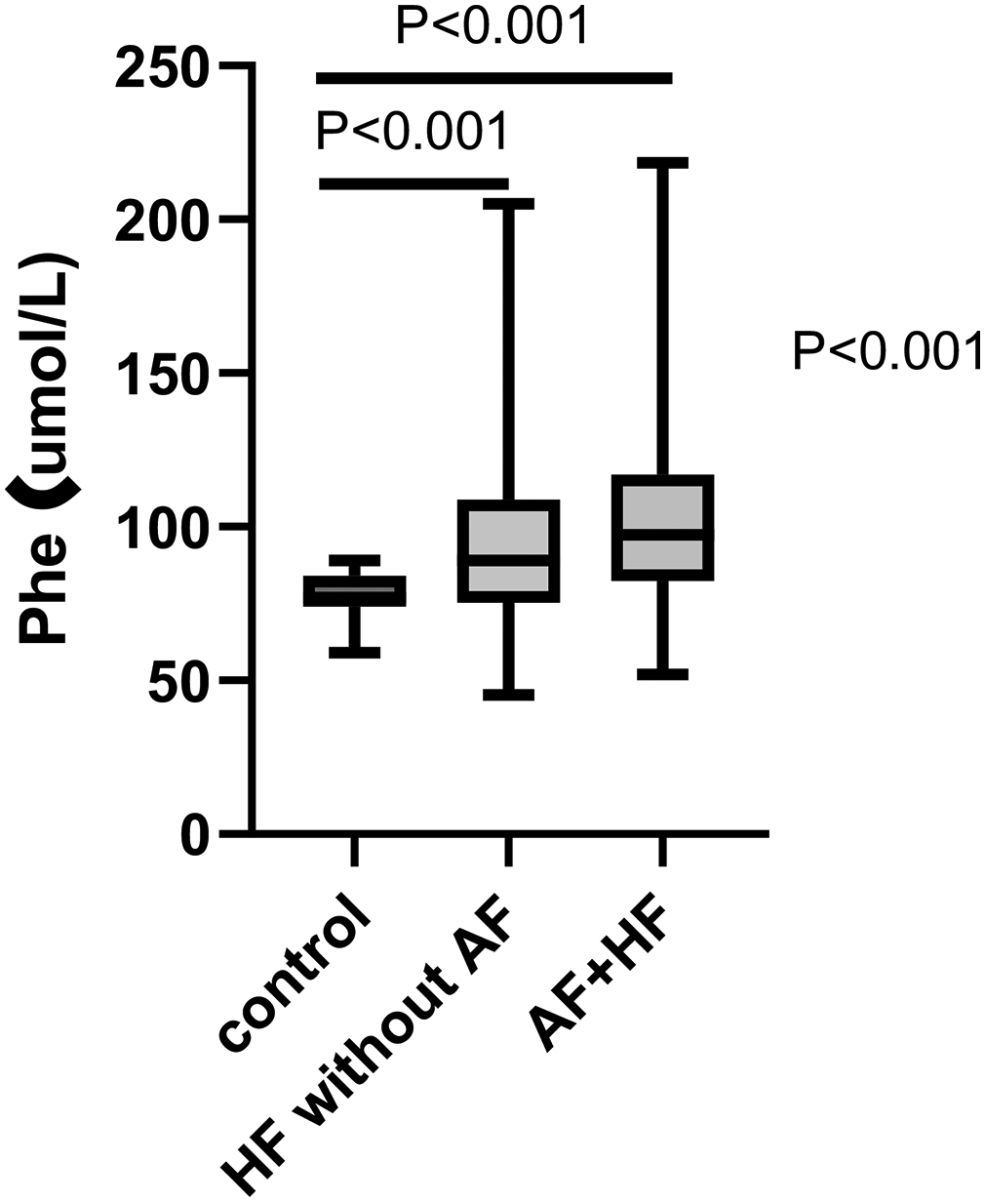
The expression of Phe value in various groups by one-way AVONA. Phe, phenylalanine; HF, heart failure; AF, atrial fibrillation.

The Phe value was gradually increased along with the control group, the HF without AF group, and the HF with AF group (ANOVA *P* < 0.001). Further analysis by LSD indicated that the Phe value was significantly higher in the HF without AF group compared to the control group (95.24 ± 28.58 umol/L vs. 77.60 ± 8.67 umol/L, *P* < 0.001) and even higher in the HF with AF group compared to the control group (102.90 ± 30.43 umol/L vs. 77.60 ± 8.67 umol/L, *P* < 0.001) (Figure 1).

Table 2 summarizes the demographic data and clinical features of HF patients in the study. Gender, age, BMI, systolic and diastolic BP with hypertension history, heart rate, smoking, drinking, CAD, hyperlipidemia with higher triglyceride and cholesterol levels (missing 2.67%), stroke, DM with abnormal glucose control (hbA1c missing 5.67%) and therapy, CKD history with lower eGFR (missing 0.67%), and alanine transaminase (ALT) (missing 0.67%) did not differ significantly among patients in the three Phe groups (*P* > 0.05). Left atrial and ventricular sizes, ejection fraction (EF), and serum NT-proBNP levels also showed no significant variation (*P* > 0.05) across the groups. However, the incidence of AF, the proportion of serious NYHA functional class (III or IV), and serum tyrosine (Tyr) levels increased while FR decreased from the low-Phe group to the intermediate-Phe group and high-Phe group (*P* < 0.05) (Table 2). Additionally, patients in the high-Phe group had higher serum hsCRP values compared to those in the low-Phe and intermediate-Phe groups (*P* < 0.05) (missing 15.67%). The use of anticoagulant drugs was more prevalent in the intermediate- and high-Phe groups than that in the low-Phe group (*P* < 0.05). The anti-HF (ACEI/ARB/ARNI, beta-blocker, calcium antagonist, digoxin, and diuretics), aspirin, and statin therapy had no significant difference among the three groups (*P* > 0.05).

**Table 2:** Baseline characteristics of HF patients according to the tertiles of Phe value.

(umol/L)	Phe ≤ 80.94	81.07 ≤ Phe ≤ 103.97	Phe ≥ 104.05	*P* value
	(*n* = 100)	(*n* = 100)	(*n* = 100)	
Demographic and clinical features				
Men, *n* (%)	77 (77.0)	76 (76.0)	81 (81.0)	0.665
Age (years)	63 ± 14	66 ± 13	65 ± 12	0.354
BMI (kg/m^2^)	24.43 ± 3.11	24.28 ± 4.86	24.42 ± 3.82	0.958
Systolic BP (mmHg)	130.12 ± 21.68	126.93 ± 21.77	124.40 ± 22.64	0.186
Diastolic BP(mmHg)	76.51 ± 15.80	76.24 ± 14.75	75.28 ± 13.97	0.828
Heart rate (bpm)	81.83 ± 17.01	80.97 ± 15.80	83.58 ± 18.93	0.554
NYHA class II, *n* (%)	51 (51.0)	39 (39.0)	28 (28.0)	0.022
III, *n* (%)	37 (37.0)	49 (49.0)	56 (56.0)	
IV, *n* (%)	12 (12.0)	12 (12.0)	16 (16.0)	
Medical history				
Smoking, *n* (%)	41 (41.0)	33 (33.0)	26 (26.0)	0.079
Drinking, *n* (%)	29 (29.0)	22 (22.0)	20 (20.0)	0.290
Atrial fibrillation, *n* (%)	15 (15.0)	23 (23.0)	32 (32.0)	0.018
Coronary disease, *n* (%)	60 (60.0)	58 (58.0)	50 (50.0)	0.321
Hypertension, *n* (%)	62 (62.0)	59 (59.0)	65 (65.0)	0.682
Hyperlipidemia, *n* (%)	7 (7.0)	9 (9.0)	3 (3.0)	0.207
Stroke, *n* (%)	3 (3.0)	8 (8.0)	4 (4.0)	0.229
Diabetes mellitus, *n* (%)	36 (36.0)	37 (37.0)	33 (33.0)	0.827
Chronic kidney disease, *n* (%)	13 (13.0)	12 (12.0)	10 (10.0)	0.797
Echocardiography and laboratory values				
Left atrial diameter (mm)	44.89 ± 6.18	45.22 ± 7.03	45.93 ± 7.12	0.542
Left ventricular: EDD (mm)	61.09 ± 7.67	61.08 ± 8.43	61.96 ± 9.06	0.697
ESD (mm)	49.25 ± 8.51	49.51 ± 9.06	50.48 ± 9.65	0.602
EF%	37.18 ± 8.39	36.83 ± 8.72	35.65 ± 8.53	0.416
Log NT-proBNP	3.24 ± 0.68	3.22 ± 0.63	3.29 ± 0.74	0.755
Triglyceride (mmol/L)	1.34 ± 0.72	1.36 ± 0.79	1.32 ± 0.58	0.925
Total cholesterol (mmol/L)	3.97 ± 1.16	4.00 ± 1.16	4.17 ± 1.23	0.425
HDL-c (mmol/L)	1.09 ± 0.27	1.10 ± 0.26	1.07 ± 0.29	0.643
LDL-c (mmol/L)	2.40 ± 0.98	2.42 ± 0.98	2.61 ± 1.05	0.287
HbA1c%	6.46 ± 1.34	6.67 ± 1.60	6.46 ± 1.22	0.500
ALT (IU/L)	26.82 ± 22.24	26.44 ± 22.93	42.87 ± 97.17	0.083
eGFR (ml/min/1.73 m^2^)	71.61 ± 27.33	65.88 ± 25.26	63.57 ± 24.26	0.078
hsCRP (mg/L)	7.53 ± 11.85	6.50 ± 11.70	22.51 ± 48.35	0.001
Tyrosine(umol/L)	63.21 ± 14.04	76.05 ± 19.64	88.65 ± 28.72	<0.001
Fisher's ratio	3.59 ± 0.96	3.46 ± 0.86	2.94 ± 0.77	<0.001
Medications				
ACEI/ARB/ARNI, *n* (%)	80 (80.0)	68 (68.0)	80 (80.0)	0.072
Beta-blocker, *n* (%)	89 (89.0)	85 (85.0)	83 (83.0)	0.468
Calcium antagonist, *n* (%)	13 (13.0)	9 (9.0)	11 (11.0)	0.665
Digoxin, *n* (%)	2 (2.0)	6 (6.0)	8 (8.0)	0.157
Diuretics, *n* (%)	56 (56.0)	62 (62.0)	51 (51.0)	0.291
Aspirin, *n* (%)	49 (49.0)	42 (42.0)	38 (38.0)	0.282
Anticoagulant, *n* (%)	19 (19.0)	34 (34.0)	33 (33.0)	0.032
Statins, *n* (%)	73 (73.0)	75 (75.0)	65 (65.0)	0.257
Hypoglycemic drugs, *n* (%)	35(35.0)	38(38.0)	32(32.0)	0.673

HF, heart failure; Phe, phenylalanine; BMI, body mass index; BP, blood pressure; NYHA, New York Heart Association; AF, atrial fibrillation; EDD, left ventricular end-diastolic diameter; ESD, left ventricular end-systolic diameter; EF, ejection fraction; NT-proBNP, N-terminal brain natriuretic peptide; HDL-c, high-density lipoprotein cholesterol; LDL-c, low-density lipoprotein cholesterol; HbA1c, glycated hemoglobin A1c; ALT, alanine transaminase; eGFR, estimated glomerular filtration rate; hsCRP, high-sensitivity C-reactive protein; ACEI, angiotensin-converting enzyme inhibitor; ARB, angiotensin receptor blocker; ARNI, angiotensin receptor neprilysin inhibitor. Fischer's ratio, the sum of valine, isoleucine, and leucine divided by the sum of phenylalanine and tyrosine.

### Univariate and multivariate analysis

Age, LAD, and serum Phe levels categorized into tertiles were identified as factors correlated with the occurrence of AF in chronic HF through univariate regression analysis (*P* < 0.05). After adjusting for age and LAD, Phe tertiles were even found to be an independent risk factor for AF in chronic HF patients (OR: 1.640; 95% CI: 1.150–2.339; *P* = 0.006) (Table 3). In the logistic regression analysis, it was revealed that the risk of AF increased by 73.9% with per 1-SD increase in log Phe levels after full adjustment (OR: 1.739; 95% CI: 1.216–2.488; *P* = 0.002). Furthermore, when comparing the highest tertile to the lowest tertile of serum Phe levels, the risk of AF increased by 3.373 times (OR: 4.373; 95% CI: 1.750–10.926; *P* = 0.002) (Table 4).

**Table 3:** Logistic regression analysis of the independent risk factors for AF in HF.

	Univariate	*P* value	Multivariate	*P* value
	OR (95% CI)		OR (95% CI)	
Gender(male)	0.938 (0.494–1.778)	0.843	–	–
Age	1.960 (1.105–3.477)	0.021	2.089 (1.152–3.789)	0.015
BMI	0.859 (0.602–1.225)	0.402	–	–
Smoking	0.893 (0.503–1.585)	0.700	–	–
Drinking	0.943 (0.500–1.779)	0.856	–	–
Diabetes mellitus	0.731 (0.411–1.302)	0.287	–	–
Hyperlipidemia	0.369 (0.083–1.636)	0.189	–	–
Hypertension	0.767 (0.445–1.322)	0.340	–	–
Coronary disease	0.338 (0.194–0.590)	<0.001	–	–
Left atrial diameter	4.477 (1.851–10.832)	0.001	4.588 (1.871–11.255)	0.001
log NT-proBNP	1.161 (0.679–1.984)	0.585	–	–
low eGFR	1.223 (0.909–1.645)	0.184	–	–
hsCRP	0.840 (0.419–1.685)	0.624	–	–
Phe tertiles	1.628 (1.158–2.287)	0.005	1.640 (1.150–2.339)	0.006

OR, odds ratio; 95% CI, 95% confidence interval. Other abbreviations are similiar to those in Table 2.

**Table 4:** Serum Phe value was associated with AF in chronic HF patients.

	Unadjusted OR	*P* value	Adjusted for Model 1 OR	*P* value	Adjusted for Model 2 OR	*P* value
Log Phe per SD	1.318 (1.008–1.723)	0.044	1.329 (1.011–1.747)	0.042	1.739 (1.216–2.488)	0.002
Phe tertiles	1.628 (1.158–2.287)	0.005	1.643 (1.162–2.322)	0.005	2.081 (1.329–3.258)	0.001
Tertile 1	1(ref)		1(ref)		1(ref)	
Tertile 2	1.693 (0.824–3.477)	0.152	1.599 (0.773–3.306)	0.205	2.180 (0.858–5.539)	0.101
Tertile 3	2.667 (1.336–5.323)	0.005	2.686 (1.336–5.401)	0.006	4.373 (1.750–10.926)	0.002

Model 1: Adjusted for age and gender.

Model 2: Adjusted for age, gender, body mass index, smoking, drinking, diabetes mellitus, hyperlipidemia, hypertension, coronary disease, left atrial diameter, log NT-proBNP, low eGFR, and hsCRP.

Continuous variables were entered per 1 SD.

OR, odds ratio. Other abbreviations are similar to those in Table 2.

### Receiver-operating characteristic curve (ROC)

**Figure 2:**
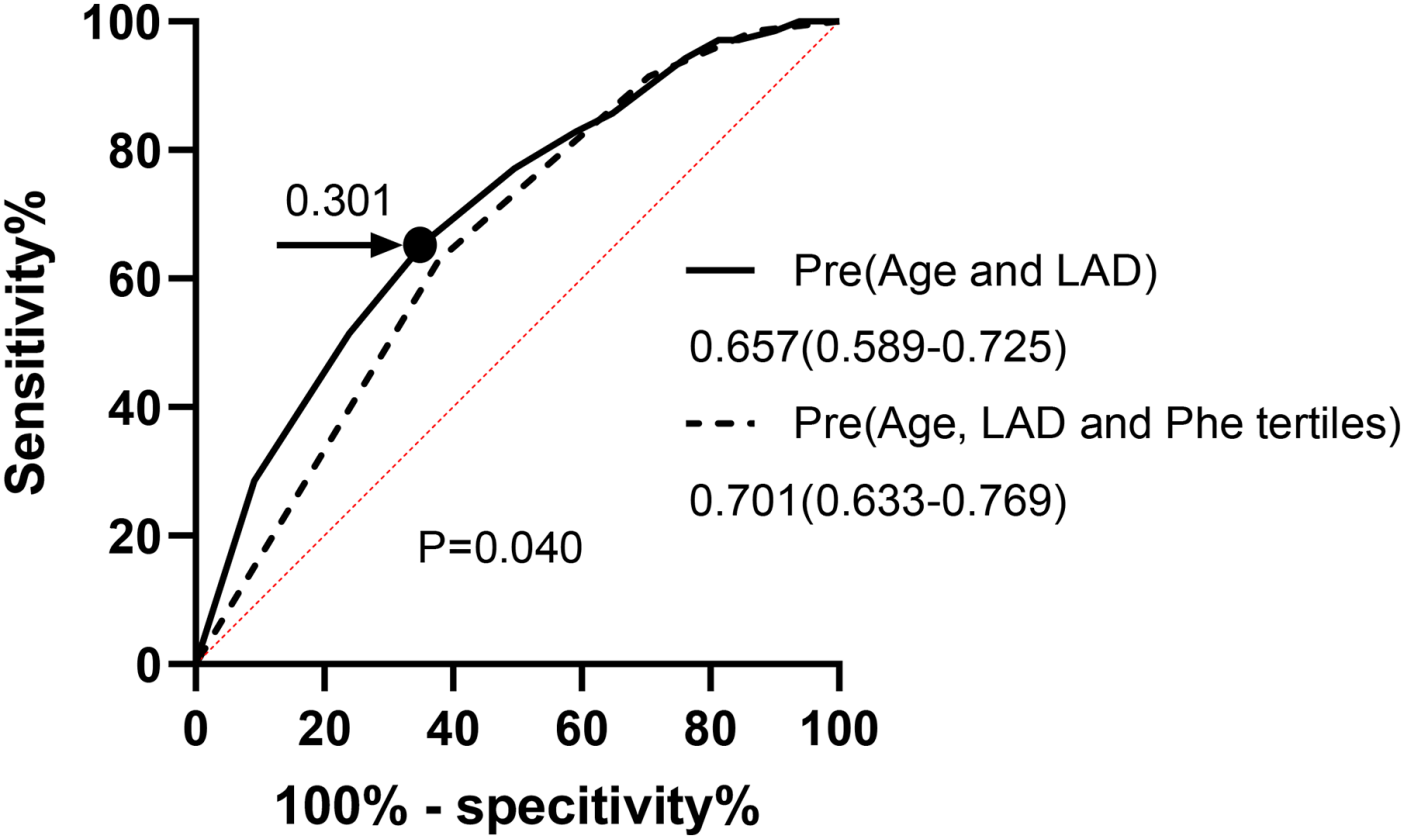
ROC curve for analysis AF in chronic HF. Pre, prediction probabilities; LAD, left atrial diameter; Phe, phenylalanine; ROC curve, receiver-operating characteristic curve; AF, atrial fibrillation; HF, heart failure.

In HF individuals, the optimal cutoff point value for predicting the presence of AF using age and LAD was determined to be 0.250, with an AUC of 0.657 (CI: 0.589–0.725; sensitivity, 62.86%; specificity, 62.17%; *P* < 0.001). Additionally, an ROC analysis was performed to evaluate the predictive abilities of age, LAD, and Phe tertiles for AF occurrence in HF patients. The optimal cutoff value for the probability was found to be 0.301, with a sensitivity of 65.71% and specificity of 64.35%. The AUC for this model was 0.701 (95% CI: 0.633–0.769; *P* < 0.001). Notably, the inclusion of Phe tertiles in the prediction model significantly improved its performance compared to the model without Phe tertiles, as demonstrated by the AUC difference analysis (0.701:0.657, *P* = 0.04) (Figure 2).

### Subgroup analysis of Phe for AF incidence in HF patients

**Figure 3:**
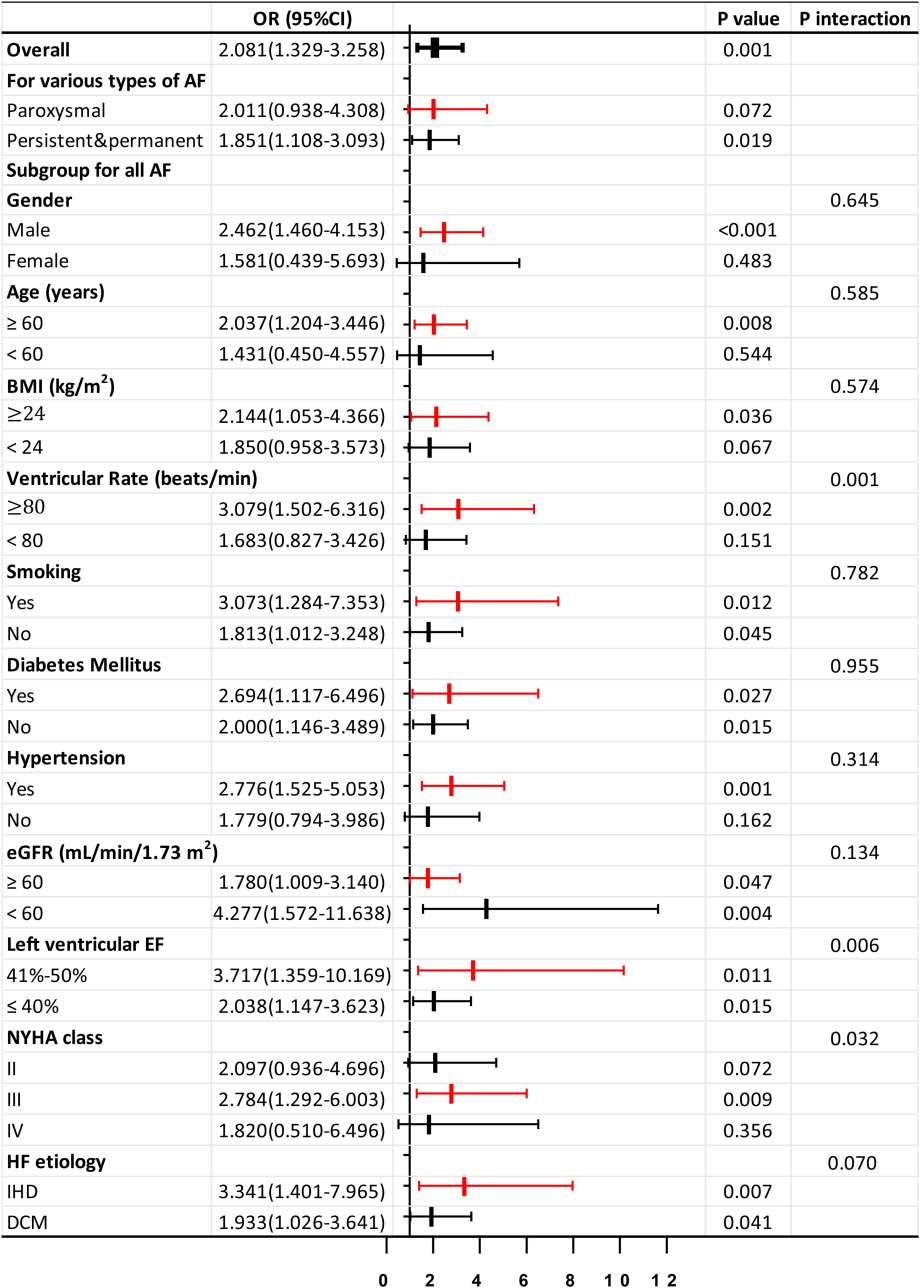
Forrest plots for subgroup analysis in HF patients. Forrest plots (adjusted) to analyze the predictive value of the Phe tertiles for AF in different subgroups of patients with HF. Adjusted for age, gender, body mass index, smoking, drinking, diabetes mellitus, hyperlipidemia, hypertension, coronary disease, left atrial diameter, log NT-proBNP, low eGFR, and hsCRP. OR, odds ratio; 95% CI, 95% confidence interval; IHD, ischemic heart disease; DCM, dilated cardiomyopathy. Other abbreviations are similar to those in Table 2.

Phe tertiles could predict AF in HF patients, especially persistent and permanent AF (*P* < 0.05). However, its significance in predicting paroxysmal AF was limited (*P* > 0.05). Further subgroup analysis using a logistic regression model indicated that Phe tertiles were more effective predictors of AF occurrence in male HF patients aged 60 years or older, with a BMI of 24 kg/m^2^ or greater, or concomitant hypertension (*P* < 0.05). This tertile was observed significantly in those with ventricular rate of ≥80 beats/min (*P* < 0.05), but not in those with a ventricular rate of <80 beats/min (*P* > 0.05, *P* interaction < 0.05). The presence of CV risk factors such as smoking, DM, renal insufficiency (eGFR < 60 ml/min/1.73 m^2^), or specific HF etiologies (dilated cardiomyopathy or ischemic heart disease, DCM, or IHD) did not influence the predictive value (*P* interaction ≥ 0.05). The predictive ability of Phe tertiles remained significant in both the HF with mildly reduced EF (HFmrEF, 41%≤EF ≤ 50%) and HF with reduced EF (HFrEF, EF ≤ 40%) subgroups (all *P* < 0.05). Furthermore, the predictive value was found to be superior in the HFmrEF group compared to the HFrEF group (*P* interaction < 0.05). This tertile was also found to be statistically significant in patients with NYHA class III (*P* < 0.05), but not in those with NYHA class II or IV (*P* > 0.05, *P* interaction < 0.05) (Figure 3).

### Correlation between serum Phe and hsCRP value

**Figure 4:**
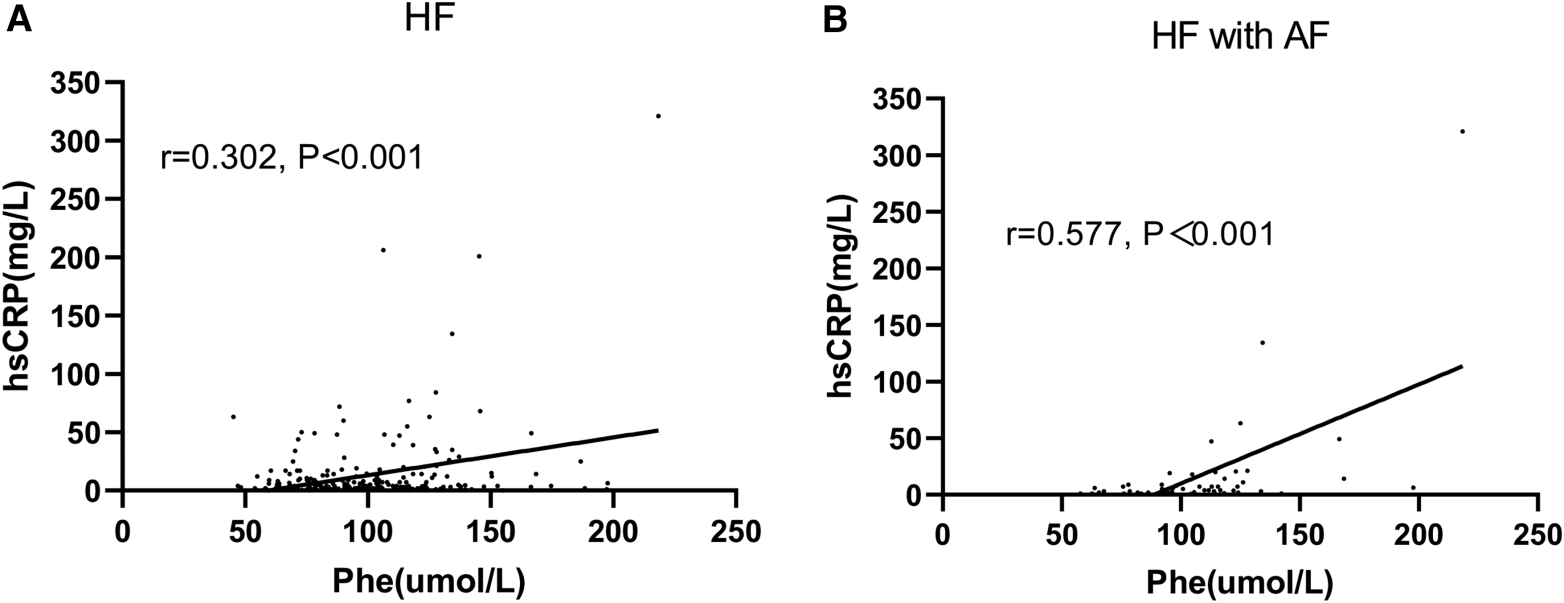
Correlation between Phe and hsCRP. Phe, phenylalanine; hsCRP, high-sensitivity C-reactive protein. **(A)** Heart failure (HF) patients; **(B)** HF patients with atrial fibrillation (AF).

The simple correlation between serum Phe levels and hsCRP values in patients with HF was found to be positive, with a correlation coefficient (*r*) of 0.302 (*P* < 0.001). This positive correlation was particularly pronounced in HF patients who also had AF, with a correlation coefficient (*r*) of 0.577 (*P* < 0.001) (Figures 4A,B). The logistic regression analysis showed that Phe tertiles were associated with hsCRP values in HF patients with AF (OR: 5.482; 95% CI: 1.346–22.320; *P* = 0.018) (Table 5).

**Table 5:** Correlation between Phe tertiles and hsCRP in HF (or with AF) subjects.

	hsCRP	*n*	Unadjusted OR	*P* value	*n*	Adjusted OR	*P* value
HF subjects	Phe tertiles	254	1.531 (1.072–2.184)	0.019	234	1.296 (0.893–1.882)	0.173
HF with AF subjects	Phe tertiles	57	2.589 (1.173–5.712)	0.018	49	5.482 (1.346–22.320)	0.018

Adjusted for age, gender, body mass index, log troponin-I, log NT-proBNP, hbA1c, LDL-c, ALT, and low eGFR.

OR, odds ratio. Other abbreviations are similar to those in Table 2.

### Prospective research of Phe value

**Figure 5:**
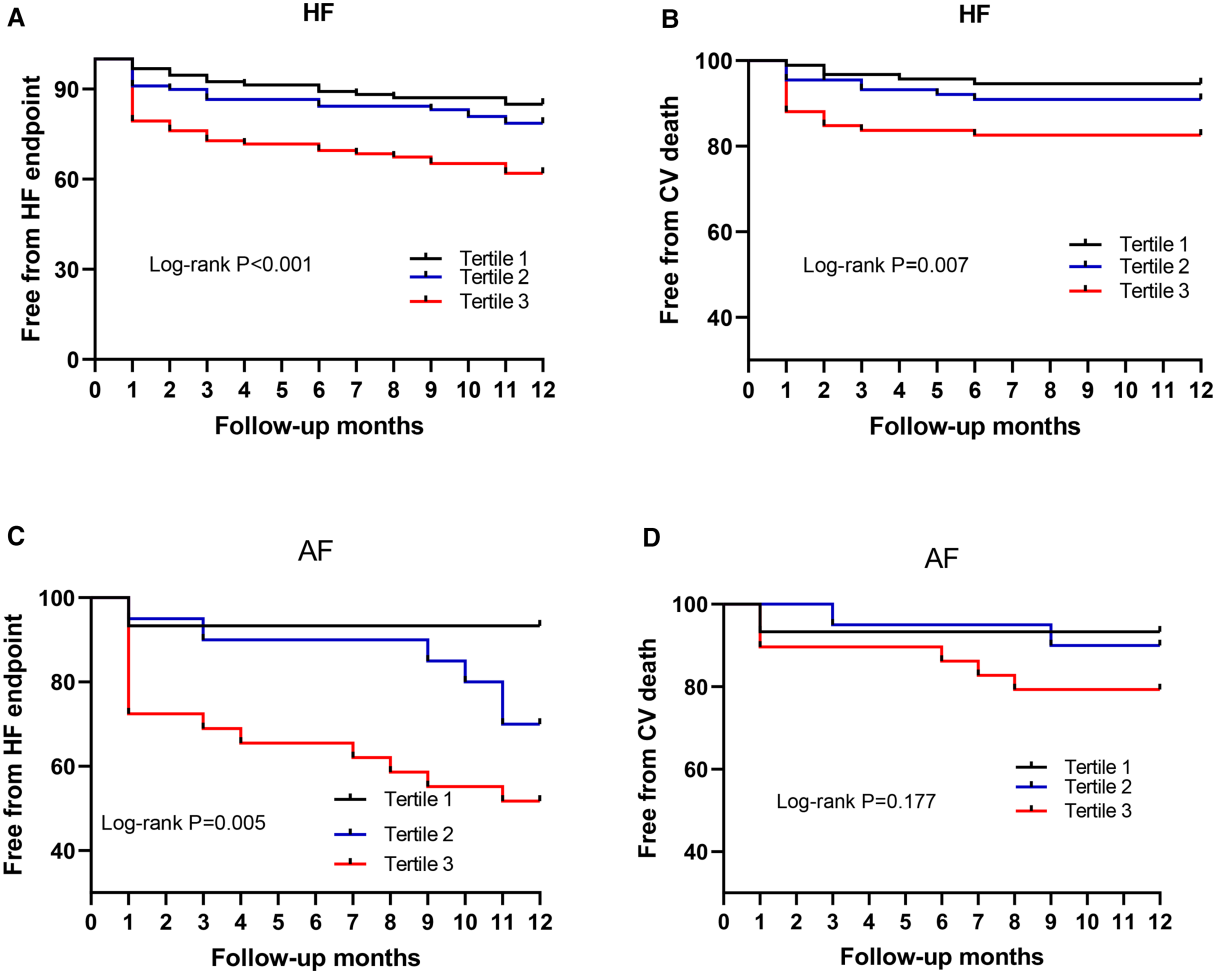
Phe value for predicting the endpoint. Phe, phenylalanine; HF, heart failure; AF, atrial fibrillation; Tertile 1, low Phe, Phe ≤ 80.94 mmol/L; Tertile 2, intermediate Phe, 81.07 mmol/L ≤ Phe ≤ 103.97 mmol/L; Tertile 3, high Phe, Phe ≥ 104.05 mmol/L. **(A**,**B)** HF patients; **(C**,**D)** HF patients with AF.

To investigate the prognostic significance of Phe levels in chronic HF, a cohort of 274 patients was followed for an average of 10.99 ± 3.00 months post-discharge, with 26 patients lost to follow-up. The composite endpoint events for HF, including CV death and first HF readmission, were selected for analysis. Of the 274 HF patients, 68 individuals (24.8%) experienced the composite endpoint events, consisting of 52 (19.0%) HF readmissions and 29 (10.6%) CV deaths. Based on Phe value tertiles, patients were categorized into three groups: Group 1 (*n* = 93, Phe ≤ 80.94 mmol/L), Group 2 (*n* = 89, 81.07 mmol/L ≤ Phe ≤ 103.97 mmol/L), and Group 3 (*n* = 92, Phe ≥ 104.05 mmol/L). The incidence of composite endpoint events for HF progressively increased from Group 1 to Group 3, with rates of 15.1%, 21.3%, and 38.0%, respectively (*P* = 0.001). Similarly, the incidence of CV death showed a gradual rise from Group 1 to Group 3, at 5.4%, 9.0%, and 17.4%, respectively (*P* = 0.025). Kaplan–Meier curves demonstrated a stepwise increase in the relationship between Phe tertiles and HF endpoint events (log-rank *P* < 0.001) (Figure 5A) and CV death (log-rank *P* = 0.007) from Group 1 to Group 3 (Figure 5B).

To further investigate the prognostic value of Phe levels in 70 chronic HF patients with AF across the Phe tertiles, a subset of 64 patients was followed for an average of 10.89 ± 3.01 months post-discharge (Group 1 to Group 3, with *n* = 15, 20, and 29, respectively), while 6 patients were lost to follow-up. Among the 64 HF patients with AF, 21 patients (32.8%) experienced the composite HF endpoint events, including 16 (25.0%) HF readmissions and 9 (14.1%) CV deaths. The incidence of composite HF endpoint events for AF patients increased incrementally from Group 1 to Group 3, with rates of 6.7%, 30.0%, and 48.3%, respectively (*P* = 0.020). The rate of CV death also displayed a progressive increase from Group 1 to Group 3, at 6.7%, 10.0%, and 20.7%, respectively; however, this difference was not statistically significant (*P* = 0.367). Kaplan–Meier curves revealed a stepwise rise in the relationship between Phe tertiles and HF endpoint events (log-rank *P* = 0.005) (Figure 5C). However, the log-rank *P* value for CV death in the Kaplan–Meier curves was 0.177 (Figure 5D).

## Discussions

Several important findings were reported in the present study. First, elevated levels of circulating Phe were linked to an increased risk of AF in patients with HF, especially the increased risk of persistent and permanent AF. Second, this association was particularly strong in older male patients with a higher BMI. The predictive value of Phe for AF was also similar in subgroups with or without risk factors such as smoking, DM, hypertension, renal insufficiency, and various causes of HF or EF levels. It is especially noteworthy in the subgroup of HFmrEF, NYHA class III, or ventricular heart rate over 80 beats/min. Third, circulating Phe values were positively correlated with serum hsCRP levels in HF with AF. Finally, the patients with high Phe levels also had a higher risk of experiencing HF-related endpoint events in the presence of AF. While prior evidence has suggested a link between Phe and HF, this study contributes unique insights by being the first to explore the role of serum Phe levels in predicting AF occurrence in HF patients. Given that AF and HF share common pathophysiological mechanisms and often coexist, understanding the relationship between Phe and AF in the context of HF could offer valuable insights for improving prevention and management strategies for these conditions.

Poor perfusion and congestion may contribute to hepatic dysfunction in HF (18–20), which reduces protein synthesis and affects AA metabolism, resulting in increased levels of free aromatic AAs, including serum Phe levels. In the present study, we showed that serum Phe value increased along with the elevated proportion of NYHA III or IV grade in HF patients and was higher than in the control group. However, AF is not causal in cardiomyopathy but is temporally associated with worsening symptoms and HF hospitalization. There is heterogeneity in the risk of secondary AF based on whether the primary cardiomyopathic process directly affects the LA or rather indirectly affects the LA through pathologic LV remodeling (21). Systemic inflammation caused by cytokines and catabolic hormones released in HF can contribute to the production of reactive oxygen species, leading to the depletion of tetrahydrobiopterin (BH_4_), a cofactor for enzymes involved in metabolizing Phe and Tyr. The resulting accumulation of Phe and Tyr may further increase serum Phe levels (22, 23). Furthermore, inflammation plays a dual role in both the development of HF and the onset of AF (24, 25). Continuous inflammation can lead to structural and electrical changes in the heart, triggering and sustaining AF (26). Notably, our study revealed that serum Phe levels were correlated with hsCRP levels in HF patients with AF. This suggests a potential link between inflammation, Phe levels, and the development of AF in the HF population.

The HF subgroup analysis further explored the prediction value of serum Phe for AF incidence in males, older, smokers, obesity, DM, hypertension, and renal insufficiency, which were also common risk factors for AF (27). These risk factors, including DM, hypertension, renal dysfunction, and aging, could disrupt Phe metabolism and reduce the availability of BH_4_ in HF (10, 28). Numerous population-based studies have shown that advancing age, gender, or elevated BMI could also increase AF incidence (29–32). Hypertension, on the other hand, leads to adverse cardiac effects such as fibrosis, cell death, and inflammation, contributing to left ventricular hypertrophy and remodeling (33). Furthermore, our analysis revealed that Phe levels may serve as a better predictor for male HF patients who are older, have a higher BMI, or suffer from hypertension. Smoking has a dose–response relationship with AF, causing structural changes in the myocardium that directly affect its conductive properties (34). Obesity, DM, and AF were interconnected conditions influenced by oxidative stress and inflammation, exacerbating atrial remodeling and creating a conducive environment for AF (35). CKD could elevate levels of angiotensin II and C-reactive protein, ultimately promoting atrial tissue damage and fibrosis, increasing the risk of AF (36–39). Left atrial enlargement and diastolic dysfunction related to AF were also more common in patients with CKD (40). The pathological changes associated with these risk factors collectively contribute to abnormalities in myocardial electrical activity, which may trigger AF development in HF patients with elevated serum Phe levels. However, our subgroup analysis demonstrated that Phe levels can predict AF not only in patients with smoking, DM, or CKD but also in those without these risk factors, suggesting that the predictive value of Phe for AF is relevant for a broader spectrum of HF patients with varying etiologies, including DCM and IHD.

Additionally, our study found that serum Phe levels were predictive of AF in both HFmrEF and HFrEF subgroups. Interestingly, the predictive value was more pronounced in the HFmrEF subgroup, aligning with the results from Sartipy U's team, who observed a progressive increase in AF with higher EF (41). This suggests that the development of AF was not necessarily linked to the worsening of HF, contradicting previous beliefs that HF exacerbates AF occurrence. Moreover, in our study, the incidence of CAD was significantly higher in the HFmrEF subgroup compared to the HFrEF subgroup (49.7% vs. 67.3%, *P* = 0.003), while other contributing factors showed no significant differences between the two groups (*P* > 0.05). This disparity in CAD rates may explain why serum Phe levels were indicative of AF risk in the HFmrEF subgroup, as CAD was known to predispose individuals to develop AF (42). Meanwhile, the findings of our study indicate that serum Phe was significantly predictive of AF occurrence in the NYHA Class III subjects, which matched the high predictive value in HFmrEF subjects. The European Heart Rhythm Association (EHRA) score, a validated tool for assessing AF symptoms, was shown to be directly correlated with baseline NYHA functional class (43). It indicated that as the functional classification deteriorates, tolerance to AF worsens, potentially explaining the higher prediction efficiency of serum Phe in HF subjects with a more severe NYHA class. In summary, plasma Phe levels hold promise as potential circulating markers for predicting AF, particularly in those with HFmrEF and NYHA class III.

Another finding in our subgroup analysis indicated that serum Phe could predict AF in HF subjects with a ventricular rate exceeding 80 beats/min. In the Get With The Guidelines HF Program, patients with lenient (<110 bpm) vs. strict (<80 bpm) resting rate control in AF fared worse (44). Therefore, it was pointed out that regardless of the size of LVEF, a heart rate of more than 80 beats/min was associated with adverse outcomes of AF, so serum Phe value had a higher detection efficiency for AF in this HF population.

Then, this research also showed that the Phe indicator had a lower prediction value for paroxysmal AF than for persistent and permanent AF in the HF subjects. This may be attributed to interference from including patients with persistent and permanent AF in the HF target group and limitations due to small sample sizes.

Our prospective study revealed that among HF patients, particularly with AF, those in the highest tertile of serum Phe levels were at a higher risk of experiencing HF-related endpoint events. Metabolomic analyses indicated that serum Phe levels exhibited the strongest correlation with HF hospitalizations, especially in elderly individuals (11). This association may be attributed to the role of Phe and Tyr as precursors of catecholamines such as epinephrine and norepinephrine, which were elevated in HF patients due to the stress response triggered by reduced cardiac output (45). Furthermore, impaired Phe metabolism and BH_4_ deficiency could lead to hemodynamic instability and impaired microcirculation, contributing to higher mortality rates among HF patients (46). The presence of AF as a comorbidity in HF patients may worsen their condition, leading to an increase in endpoint events and elevated Phe levels. Although the difference in survival rates based on Phe tertiles was not significant among AF patients in our study due to the limited sample size, the association remains clinically relevant for HF patients. We aim to enhance prognostic insights by expanding our research to include a larger cohort of HF patients with AF. The broader study population may provide a more robust foundation for exploring the prognostic significance of serum Phe levels in HF patients with AF, potentially uncovering more meaningful clinical implications for patient care.

Last but not least, we should not ignore the secondary causes of AF, independently from chronic HF. In particular, AF in the young may be precipitated by hyperthyroidism; lifestyle factors such as endurance sports, alcohol consumption, and even smoking; cardiomyopathy; and channelopathies. The development of AF is often triggered by hyperthyroidism, leading to tachycardia and increased cardiac load due to hyperkinetic circulation (47). Engaging in endurance exercise can increase the likelihood of developing AF by two to ten times (48). Smoking also increases the risk of AF by more than two times, and individuals who quit smoking tend to reduce the incidence of AF (49). Although most HCM athletes are asymptomatic, over 95% of cases exhibit electrocardiogram abnormalities, and electrical abnormalities may manifest several years earlier than structural abnormalities, which is a significant cause of sudden cardiac death (50). Long QT syndrome (LQTS), resulting from abnormal ion channel function, may also impact atrial electrophysiology and is linked to the risk of AF. The prior research identified a notable association between the long LQT3 genotype and the risk of early-onset AF (51). Therefore, these factors should be taken into consideration in the management of AF conditions.

## Conclusions

Circulating Phe value was the independent risk factor for the prediction of AF in chronic HF. High Phe levels could indicate inflammatory disorder in HF with AF patients. In addition, increased Phe value was associated with the poor HF endpoint events of HF patients with AF. Phe could be a valuable indicator of AF in HF.

## Data Availability

The original contributions presented in the study are included in the article/Supplementary Material; further inquiries can be directed to the corresponding authors.
